# Association between olfactory dysfunction and mood disturbances with objective and subjective cognitive deficits in long-COVID

**DOI:** 10.3389/fpsyg.2023.1076743

**Published:** 2023-02-02

**Authors:** Tania Llana, Marta Mendez, Sara Garces-Arilla, Vanesa Hidalgo, Magdalena Mendez-Lopez, M.-Carmen Juan

**Affiliations:** ^1^Department of Psychology, Faculty of Psychology, University of Oviedo, Oviedo, Spain; ^2^Neuroscience Institute of Princedom of Asturias (INEUROPA), Oviedo, Spain; ^3^Instituto de Investigación Sanitaria del Principado de Asturias (ISPA), Av. del Hospital Universitario, Oviedo, Spain; ^4^Instituto Universitario de Automática e Informática Industrial, Universitat Politècnica de València, Valencia, Spain; ^5^Department of Psychology and Sociology, University of Zaragoza, Zaragoza, Spain; ^6^Laboratory of Social Cognitive Neuroscience, Department of Psychobiology, University of Valencia, Valencia, Spain; ^7^IIS Aragon, Zaragoza, Spain

**Keywords:** long-covid, memory, cognition, anxiety, depression, olfactory dysfunction

## Abstract

**Background and purpose:**

The coronavirus disease 2019 (COVID-19) has been associated with olfactory dysfunction. The persistent symptoms of anosmia or hyposmia were associated in previous studies with the development of memory impairment and mood disturbances. We aimed to investigate the association between the chronicity of reported olfactory dysfunction and subjective and objective cognitive performance in long-COVID patients and to explore whether their emotional symptoms are related to their cognition.

**Methods:**

One hundred twenty-eight long-COVID participants were recruited. Reported symptomatology, subjective memory complaints, anxiety and depression symptomatology, and trait-anxiety were assessed. Subjective memory complaints and mood disturbances were compared among groups of participants with olfactory dysfunction as an acute (AOD), persistent (POD), or nonexistent (NOD) symptom. Seventy-six of the volunteers also participated in a face-to-face session to assess their objective performance on tests of general cognitive function and verbal declarative memory. Objective cognitive performance and mood disturbances were compared among the AOD, POD, and NOD groups.

**Results:**

The subjective memory complaints and the anxiety and depression symptoms were similar among the groups, but the score in general cognitive function was lower in the participants with symptoms of acute olfactory dysfunction than in those with no olfactory symptoms at any time. Participants’ memory complaints were positively related to their emotional symptoms. The relationship between depressive symptomatology and memory complaints interacted with the olfactory dysfunction, as it only occurred in the participants without symptoms of olfactory dysfunction. Depressive symptomatology and acute olfactory symptoms were negatively associated with general cognitive function and delayed memory performance. The months elapsed from diagnosis to assessment also predicted delayed memory performance. Anxious symptomatology was negatively associated with the immediate ability to recall verbal information in participants who did not present olfactory dysfunction in the acute phase of the infection.

**Conclusion:**

Olfactory dysfunction in the acute phase of the infection by COVID-19 is related to cognitive deficits in objective tests, and mood disturbances are associated with self-reported and objective memory. These findings may contribute to further understanding the neuropsychological and emotional aspects of long-COVID.

## 1. Introduction

The novel coronavirus severe acute respiratory syndrome 2 (SARS-CoV-2), from which coronavirus disease 2019 (COVID-19) comes, has had an important impact at multiple levels (Hossain et al., 2020; Nicola et al., 2020). Following the World Health Organization (WHO), some of the most frequent symptoms in the acute phase of COVID-19 are fever, cough, tiredness, headache, and anosmia/dysgeusia (World Health Organization, 2021a), and these symptoms frequently disappear over time. However, around 10–20% of the people who had COVID-19 presented persistent symptoms (Greenhalgh et al., 2020; Carod-Artal, 2021). Long-COVID has been defined by WHO as a condition that occurs in individuals with a history of probable or confirmed SARS-CoV-2 infection, usually three months from the onset of COVID-19, with symptoms that last for at least two months and cannot be explained by an alternative diagnosis (World Health Organization, 2021b; Lai et al., 2022). Long-COVID is also known as long-haul COVID-19, post-COVID-19, post-acute COVID-19 and chronic COVID-19. Its aetiology is still unknown, although three principal theories are currently proposed: viral persistence, SARS-CoV-2 superantigen-mediated activation of the immune system, and autoimmunity (Brodin et al., 2022).

Olfactory dysfunction is a frequent symptom reported by long-COVID patients (Doty, 2022; Kay, 2022) and may have different causes (Doty, 2022). These include: (i) inflammation, infection and damage of the olfactory clef, the olfactory mucosa, and olfactory neuroepithelium, which could cause overreactive immune responses within the brain; (ii) downregulation of olfactory receptor proteins on the cilia of olfactory receptor cells; (iii) long-lasting damage of nervous system networks devoted to olfactory function, including the olfactory bulb, brain cells and capillary endothelial cells, in some cases, as a result of massive activation of macrophages and release of cytokines (Xydakis et al., 2021; Doty, 2022). High viral load in the nasal cavity and infected non-neuronal cells in the olfactory sensory epithelium can produce fast-onset anosmia caused by inflammation and with rapid remission (Doty, 2022). However, peripheral or central mechanisms may also be responsible for long-term olfactory disturbances. Cell damage or death of non-neuronal cells in the sensory epithelium, especially when basal cells are extensively damaged, downregulation of olfactory receptor genes (Zazhytska et al., 2022), and damage of olfactory sensory neurons could result in long-lasting olfactory disturbances (Doty, 2022). SARS-CoV-2 virus cannot invade olfactory sensory neurons using the transneuronal route (Brann et al., 2020) but it can enter the olfactory bulbs and affect the brain through transcribriform or vascular routes. In this way, it can infect many types of glial cells (Vargas et al., 2020), causing microglial and astrocytic activations that could affect synapses and neurons, as well as neurogenesis. The latter was altered in the hippocampus of patients and hamsters infected by the virus (Soung et al., 2022). Interestingly, chronic inflammation and suppressing hippocampal neurogenesis are associated with memory impairment and mood disorders, such as depression and anxiety (Chesnokova et al., 2016). Emotion and memory function might be affected by the suppression of hippocampal neurogenesis. The release of pro-inflammatory cytokines and the activation of microglia reduce adult hippocampal neurogenesis, which, in turn, causes mood and cognitive disturbances typically observed in chronic inflammatory disorders (Chesnokova et al., 2016). Damage to the olfactory bulb was demonstrated in long-COVID patients associated with long-term olfactory dysfunction (Frosolini et al., 2022). This damage might extend to proximal and connected regions, affecting the limbic system and, consequently, impairing emotional and memory networks (Díez-Cirarda et al., 2022; Goehringer et al., 2022; Kay, 2022; Martini et al., 2022). In fact, volume reduction and degeneration of brain areas connected to the olfactory bulb, such as the hippocampus, parahippocampal cortex, and the amygdala, with an important role in memory and emotional processing, were observed in brain scans of subjects who suffered mild COVID-19 infection (Douaud et al., 2022).

Long-term olfactory loss in long-COVID patients is associated with the development of neuropsychological alterations, including memory impairment (Kay, 2022) and mood disturbances. Previous studies which assessed memory in long-COVID patients have mainly explored declarative verbal memory (Llana et al., 2022b). These studies have found impairment in verbal learning, verbal short-term memory and verbal long-term memory assessed with neuropsychological tests such as the Rey Auditory Verbal Learning Test (RAVLT), in both hospitalised and non-hospitalised adults (Crivelli et al., 2022; García-Sánchez et al., 2022). Declarative verbal memory is essential to remember ongoing experiences and to learn new information about facts and events (Tulving and Markowitsch, 1998). Mood disturbances were related to memory impairment in previous research conducted in non-COVID population. In this sense, objective memory dysfunction assessed with neuropsychological tests was significantly associated with anxiety and depression (Arbabi et al., 2015). Also, self-reported memory complaints, assessed by different questionnaires in which participants report every-day subjective memory function, were also associated with anxiety and depression symptomatology in healthy subjects without objective memory impairment (Balash et al., 2013). Regarding declarative verbal memory, studies which objectively assessed this type of memory have found an association between declarative memory impairment and mood disorders (Biringer et al., 2007; Chepenik et al., 2012; Engelmann et al., 2020). In long-COVID population, many studies assessing the relevance of clinical symptoms have found an association between subjective memory complaints and depressive feelings (Titze de Almeida et al., 2022) or the presence of anxiety and depression (Almeria et al., 2020; Cysique et al., 2022). The study by Voruz et al. (2022) found that memory and mood disturbances in long-COVID patients who suffered a mild or moderate disease correlated with hyposmia and/or anosmia, suggesting that chronic olfactory dysfunction could be related to the impairment of the limbic system. In this way, declarative verbal memory, a memory system mainly supported by the medial temporal lobe including the hippocampus and other limbic system structures (Catani et al., 2013), could be more impaired in long-COVID patients than other memory systems non-related to the limbic system function, such as procedural memory (Llana et al., 2022a). Olfactory dysfunction was associated not only with subjective memory complaints but also with objective verbal (Damiano et al., 2022) and episodic (Delgado-Alonso et al., 2022) memory performance, as well as with executive dysfunction and anxiety, but not depression (Delgado-Alonso et al., 2022). Specifically, the study of Cecchetti et al. (2022) found an association between dysgeusia and hyposmia during acute COVID-19 and increased vulneravility in declarative memory over time. However, more research is needed to better understand the interaction between chronic olfactory disturbances and memory and mood disturbances in long-COVID patients.

Based on the above issues, we hypothesised that there is an association between the chronicity of olfactory dysfunction and memory impairment, considering both self-rated and objective performance measures in long-COVID patients. Also, this memory impairment is predicted by negative emotional states. To address these hypotheses, we first considered as dependent variables the subjective memory complaints, anxiety and depression symptoms, and trait-anxiety in a sample of long-COVID patients divided into groups based on the presence of olfactory dysfunction as an acute (AOD), persistent (POD), or nonexistent (NOD) symptom. This division aims to distinguish initial anosmic/hyposmic patients from long-term anosmic/hyposmic patients. We compared the scores in these variables among the groups. Age, educational and socio-economic status and ventilatory assistance were also considered to control for their association with the dependent variables. Months from diagnosis to assessment, symptoms of anxiety and depression, and trait-anxiety were considered as independent variables predicting the memory complaints and considering the olfactory dysfunction as a factor that might interact with these predictions. Second, we further examined whether the objective memory performance in a hippocampal-dependent task, evaluated with a declarative verbal memory test, and cognitive function, assessed by a cognitive screening test, could differ among groups. We also determined the predictive value of the abovementioned independent variables in the objective memory performance and the contribution of the olfactory dysfunction to these predictions.

## 2. Materials and methods

### 2.1. Participants

This cross-sectional study was conducted in Spain between April 1 and September 23, 2022. Information about the study was disseminated *via* long-COVID associations and media.

This study was conducted in compliance with the European Community Council Directive 2001/20/EC and the Helsinki Declaration for biomedical research involving humans and approved by the ethics committee (UPV P04_16_02_2022). The experimental data were collected after obtaining written informed consent from each participant.

One hundred fifty-one individuals with long-COVID volunteered to participate. The study was finally completed by 132 of them. Four participants were excluded from the final sample because they did not meet the eligibility criteria. The final sample included 128 participants. Criteria for inclusion met with the standards of WHO definition of long-COVID (World Health Organization, 2021b) and were as follow: (1) History of probable or confirmed by RT-PCR or antigen tests SARS-CoV-2 infection at last tree months before the inclusion in the study. Probable SARS-CoV-2 infection refers to those symptomatic patients with suspected infection in their medical histories who did not undergo testing, as PCR testing or antigen tests were restricted to those who were more severely unwell early in the pandemic; (2) SARS-CoV-2 infection severity ranging from mild clinical symptoms without respiratory distress to severe respiratory distress with hospitalisation; (3) Symptoms temporally related to the SARS-CoV-2 infection which extend beyond 3 months from the onset of COVID-19 and last for at least 2 months and which cannot be explained by an alternative diagnosis. These symptoms can include at least two of the following manifestations: sensory changes (affecting olfactory, gustatory and/or visual function), fatigue, shortness of breath, fever, headache, myalgia, sleep disturbances, brain fog [concentration, memory, and executive function difficulties, which describes the feeling of being mentally slow, fuzzy, or spaced out, affecting the ability to think or concentrate (Asadi-Pooya et al., 2022)], or emotional disorders (mood and/or anxiety disorder); and (4) Native Spanish speakers or high proficieny in Spanish.

Exclusion criteria included: (1) Any cognitive complaint before COVID-19; (2) Past or present neurological disorder potentially associated with cognitive impairment and sensory impairment; (3) Present or previous severe psychological or psychiatric disorder; and (4) Uncontrolled medical conditions associated potentially biassing cognitive assessments.

### 2.2. Measurements and procedure

All participants completed the questionnaires described in section 2.2.1 online, and 76 of them comprised a non-probability subsample of individuals who voluntarily participated in an additional face-to-face session described in section 2.2.2.

#### 2.2.1. On-line assessment

On-line assessment was performed using questionnaires that were sent out *via* email. Questions were presented in three Survey Monkey questionnaires for participants to complete at home without a set time or order. One questionnaire included items to collect sociodemographic data, as well as main symptoms using the Long COVID Pre Assessment Questionnaire (National Health Service, 2021). A further questionnaire assessed subjective memory with the Memory Failures in Every-day life (MFE; Sunderland et al., 1984). Finally, depressive and anxiety symptomatology were assessed using questions from the Goldberg Anxiety and Depression Scale (GADS; Goldberg et al., 1988) and trait-anxiety items of the brief version of the State–Trait Anxiety Inventory (STAI; Spielberger et al., 1970), which were condensed into a separate questionnaire. A more detailed description of these questionnaires is provided below.

Long-COVID symptomatology (including olfactory dysfunction) was collected using a Spanish adaptation of the National Health Service (NHS) Long COVID Pre Assessment Questionnaire version 3 (National Health Service, 2021). In this questionnaire, participants reported whether olfactory dysfunction was present both in the acute phase of the infection and at the time of assessment. This information was used to classify the participants into three groups: AOD group, which comprised individuals with olfactory dysfunction only in the acute phase of the disease (within 1 week post-infection); POD group, which included individuals presenting olfactory dysfunction from the initial phase to the time of assessment (3–30 months post-infection); and NOD group, which gathered individuals without symptoms of olfactory dysfunction at any time.

Subjective memory complaints were assessed with MFE (Sunderland et al., 1984). We used the Spanish version of Montejo et al. (2012). The scale is composed of 28 items in which participants report the frequency of memory failures on a 3-point Likert scale ranging from 0 to 2 (maximum score: 56; Cronbach’s alpha value in this study was 0.93). This questionnaire has three factors: memory of activities (MFEA; maximum score: 20), recognition (MFER; maximum score: 12), and communication monitoring (MFEC; maximum score: 24; Montejo et al., 2012).

Anxiety and depression symptomatology were assessed using the GADS (Goldberg et al., 1988). We employed a Spanish version of GADS (Monton et al., 1993) with 18 items (9 for anxiety and 9 for depression; the maximum score of each subscale is 9 with higher scores indicating more anxiety and/or depression). In this study, we obtained a Cronbach’s alpha of 0.77. The stable tendency to attend to, experience, and report negative emotions (Gidron, 2013) was also measured by the trait-anxiety items of the brief version of the STAI (Spielberger et al., 1970), developed by Buela-Casal and Guillen-Riquelme (2017). This version of the scale presents 4 items of trait-anxiety (STAI-T). Items are rated on a 4-point Likert scale ranging from 0 to 3 (the maximum score is 12, with higher scores indicating more trait-anxiety). Cronbach’s alpha value in this study was 0.74.

#### 2.2.2. Face-to-face assessment

An individual session in the university facilities was conducted to assess the participants’ current level of general cognitive function and objective declarative episodic memory performance (*n* = 76).

The Spanish Version 8.1 of the Montreal Cognitive Assessment scale (MoCA; Nasreddine et al., 2005) was used to obtain a score of the overall level of cognitive abilities (maximum score: 30; cognitive impairment: < 26).

In addition, a Spanish adaptation of the Paired-Associate Learning (PAL) from the Wechsler Memory Scale (WMS-III; Wechsler, 1997) was used to assess episodic verbal memory. The PAL task presents 8 pairs of words with no semantic relation. Participants have four recall tests to learn the maximum number of pairs. In each of the tests, the researcher provides the first word of the pair to the participant, who must say the word that accompanied it. The four learning tests provide an immediate recall score (PALIR; maximum: 32). Delayed recall (PALDR) and delayed recognition (PALDRe) are also evaluated 20–30 min later (maximum scores: 8 and 24, respectively).

### 2.3. Statistical analyses

Kolmogorov–Smirnov and Levene tests were performed to examine the normal distribution and homogeneity of the variances of the main variables of the data set, respectively. Most of the variables followed a non-normal distribution, so the group comparisons and correlations between the variables were calculated using non-parametric tests.

Kruskal-Wallis tests were used to compare the scores of the MFE, GADS, STAI, MoCA, and PAL among the AOD, POD, and NOD groups. Post-hoc multiple comparisons with Bonferroni correction were performed when significant group effects were found.

Hierarchical multiple regression was carried out to explore whether the criterion variable (i.e., MFE, MoCA or PAL) was predicted by the independent variable (i.e., months from diagnosis to assessment, symptoms of anxiety, symptoms of depression or trait-anxiety). The factor Group (i.e., AOD, POD, or NOD) was separately considered an interaction term to determine whether the relationship between the predictor variables and the criterion variables was different as a function of the olfactory dysfunction as an acute or persistent symptom. The AOD group, POD group, and NOD group were each operationalised as dichotomic variables for regression analyses. In each of the three variables, participants meeting the criteria for inclusion in the group were coded as 1, and participants who did not meet the criteria for inclusion in the group were coded as 0. The following control variables were included in the analyses as covariates: age, educational and socio-economic status, and ventilatory assistance. Using the ENTRY method, the covariates were entered as predictors in the first block. Then, the independent variable (i.e., months from diagnosis to assessment, symptoms of anxiety, symptoms of depression or trait-anxiety) and the Group (i.e., AOD, POD, or NOD) were entered as predictors in the second block; and the independent variable, the Group, and the variable computed by their interaction were entered as predictors in the third block.

Statistical analyses were performed using the IBM SPSS Statistics, Version 26 (IBM Corp.). The level of statistical significance was set at *p* < 0.05.

## 3. Results

### 3.1. Demographic and clinical characteristics

The characteristics of the final sample (*n* = 128) are described in Table 1, which includes, among other aspects, the subjective socio-economic status reported through the scale by Adler et al. (2000), annual income (consisting of one item that was rated on a 5-point scale ranging from 10 to 50 thousand euros), body mass index, hospital admission and level of respiratory support during the acute phase of COVID-19, and long-COVID symptoms (National Health Service, 2021). The characteristics of the subsample that underwent face-to-face assessment are described in Table 2, which includes the same aspects considered in Table 1. The percentage of participants with RT-PCR or antigen tests confirmed SARS-CoV-2 was 85.9% in the full sample and 90.8% in the subsample. The proportion of participants with RT-PCR or antigen tests confirmed SARS-CoV-2 was similar among groups (Tables 1, 2). The full sample and the subsample were comparable in demographic and clinical characteristics (all *P*s > 0.149; see Supplementary File S1).

**Table 1:** Demographic information and clinical characteristics of the full sample related to the COVID history in the AOD, POD, and NOD groups.

	Full sample	AOD group	POD group	NOD group	*P*
	(N = 128)	(*n* = 22)	(*n* = 32)	(*n* = 74)	
Sex (F, M; M%)	114, 14; 10.9%	20, 2; 9.1%	30, 2; 6.3%	64, 10; 13.5%	.521^b^
Age (Years)^a^	45.50 (40–51)	44 (39.5–52.75)	44 (40–47.75)	47 (40.5–52)	.202^c^
SES^a^	7 (6–8)	8 (7–8.25)	7 (6–8)	6 (5–8)	.007^c^
Annual income^a^	30 (20–40)	35 (27.5–50)	25 (20–37.5)	30 (20–40)	0.045 ^c^
Handedness (*n*, %)					.223^b^
Right-hand	118, 92.2%	20, 99.9%	32, 100%	66, 89.2%	
Left-hand	6, 4.7%	2, 9.1%		4, 5.4%	
Ambidextrous	4, 3.1%			4, 5.4%	
Ethnicity (*n*, %)					0.837 ^b^
White	123, 96.1%	21, 95.5%	32, 100%	70, 94.6%	
Mixed ethnic groups	1, 0.8%			1, 1.4%	
Bla., Lat., Car., Afr.	3, 2.3%	1, 4.5%		2, 2.7%	
Prefer not to say	1, 0.8%			1, 1.4%	
Months^d^	17 (11–21; 3–30)	18 (12,5–25; 4–30)	18 (10.75–21; 3–29)	16 (11–21; 3–30)	0.536 ^c^
BMI^a^	24.85 (21.82–29.39)	26.97 (21,98–29.27)	25,12 (21.67–30,72)	23.71 (21.86–29.53)	0.685 ^c^
Acute phase of COVID					
Confirm. Test	110, 85,9%	21, 95,5%	30, 93,8%	59, 79,7%	.060^b^
Hospit. (*n*, %)	34, 26.6%	4, 18.2%	7, 21.9%	23, 31.1%	.382^b^
Vent. assist. (*n*, %)					.198^b^
Not applicable	104, 81.3%	17, 77.3%	28, 87.5%	59, 79.7%	
Intubated	6, 4.7%			6, 8.1%	
Enhanced RS	18, 14.1%	5, 22.7%	4, 12.5%	9, 12,2%	
Long-COVID symptoms					
Sense of taste					<.001^b^
Ageusia (*n*, %)	23, 18%	2, 9.1%	18, 56.3%	3, 4.1%	
Metal. taste (*n*, %)	22, 17.2%	5, 22.7%	8, 25%	9, 12.2%	
Fatigue (*n*, %)	122, 95.3%	20, 90.9%	31, 96.9%	71, 95.9%	.550^b^
Brain fog (*n*, %)	120, 93.8%	21, 95.5%	29, 90.6%	70, 94.6%	.693^b^
Lack concent. (*n*, %)	127, 99.2%	22, 100%	32, 100%	73, 98.6%	.692^b^
Sleep disturb. (*n*, %)	104, 81.3%	19, 86.4%	28, 87.5%	57, 77%	.356^b^
Nightmares (*n*, %)	61, 47.7%	12, 54.5%	14, 43.8%	35, 47.3%	.734^b^
Rec. fevers (*n*, %)	35, 27.3%	8, 36.4%	8, 25%	19, 25.7%	.579^b^
Headache (*n*, %)	89, 69.5%	18, 81.8%	25, 78.1%	46, 62.2%	.101^b^
Vis. disturb. (*n*, %)	90, 70.3%	11, 50%	25, 78.1%	54, 73%	.063^b^
Myalgia (*n*, %)	108, 84.4%	20, 90.9%	26, 81.3%	62, 83.8%	.616^b^
ED Diagn. (*n*, %)	22, 17.2%	4, 18.2%	6, 18.8%	12, 16.2%	.942^b^

a Data are shown as median (first quartile – third quartile).

b Pearson chi-squared test.

c Kruskal-Wallis test.

d Data are shown as median (first quartile – third quartile; minimum – maximum).

All the participants had > 12 years of education. SES = subjective educational and socio-economic status (scale range from 1 to 10 points). Annual income is reported on a 5-point scale (range from 10 to 50 thousand euros). Bla., Lat., Car., Afr. = Black, Latino, Caribbean or African; Months = months from diagnosis to assessment; BMI = body mass index; Confirm. Test = SARS-CoV-2 confirmed with antigen or PCR test; Hospit. = Hospitalisation; Vent. assist. = ventilatory assistance; RS = respiratory support; Metal. = metallic; Concent. = concentration; Disturb. = disturbance; Vis. = visual; Rec. = recurrent; ED diagn. = emotional disorder diagnosis based on clinical judgement (mood and/or anxiety disorders according to DSM-5 classification).

**Table 2:** Demographic information and clinical characteristics of the face-to-face assessed subsample related to the COVID history in the AOD, POD, and NOD groups.

	Subsample	AOD group	POD group	NOD group	*P*
	(N = 76)	(*n* = 13)	(*n* = 19)	(*n* = 44)	
Sex (F, M; M%)	68, 8; 10.5%	12, 1; 7.7%	18, 1; 5.3%	38, 6; 13.6%	.571^b^
Age (Years)^a^	46 (40–51)	41 (38–47)	43 (40.5–47)	47 (41–53)	.086^c^
SES^a^	6 (5–8)	8 (7–8)	6 (5.5–7.5)	6 (5–7)	.047^c^
Annual income^a^	30 (20–40)	30 (30–50)	30 (20–30)	30 (20–40)	.232^c^
Handedness (*n*, %)					.433^b^
Right-hand	69, 90.8%	12, 92.3%	19, 100%	38, 86.4%	
Left-hand	4, 5.3%	1, 7.7%		3, 6,8%	
Ambidextrous	3, 3.9%			3, 6.8%	
Ethnicity (*n*, %)					.521^b^
White	73, 96.1%	12, 92.3%	19, 100%	42, 95.5%	
Mixed ethnic groups					
Bla., Lat., Car., Afr.	3, 3.9%	1, 7.7%		2, 4.5%	
Prefer not to say					
Months^d^	17 (11–20; 3–30)	20 (18–25; 4–30)	17 (7.5–19; 3–29)	16 (11.5–19; 4–30)	.058^c^
BMI^a^	25.53 (21.89–31.08)	27.59 (22.84–30.04)	24.86 (20.18–30.28)	24.92 (22.14–31.18)	.520^c^
Acute phase of COVID					
Confirm. Test	69, 90,8%	12, 92,3%	18, 94,7%	39, 88,6%	.728^b^
Hospit. (*n*, %)	17, 22.4%	1, 7.7%	2, 10.5%	14, 31.8%	.067^b^
Vent. assist. (*n*, %)					.302^b^
Not applicable	64, 84.2%	11, 84.6%	18, 94.7%	35, 79.5%	
Intubated	5, 6.6%			5, 11.4%	
Enhanced RS	7, 9.2%	2, 15.4%	1, 5.3%	4, 9.1%	
Long-COVID symptoms					
Sense of taste					<.001^b^
Ageusia (*n*, %)	15, 63.2%	1, 7.7%	13, 68.4%	1, 2.3%	
Metal. taste (*n*, %)	13, 17.1%	4, 30.8%	4, 21.1%	5, 11.4%	
Fatigue (*n*, %)	72, 94.7%	13, 100%	18, 94.7%	41, 93.2%	.626^b^
Brain fog (*n*, %)	70, 92.1%	12, 92.3%	17, 89.5%	41, 93.2%	.882^b^
Lack concent. (*n*, %)	75, 98.7%	13, 100%	19, 100%	43, 97.7%	.692^b^
Sleep disturb. (*n*, %)	62, 81.6%	11, 84.6%	16, 84.2%	35, 79.5%	.866^b^
Nightmares (*n*, %)	37, 48.7%	8, 61.5%	7, 36.8%	22, 50%	.376^b^
Rec. fevers (*n*, %)	18, 23.7%	6, 46.2%	4, 21.1%	8, 18.2%	.109^b^
Headache (*n*, %)	49, 64.5%	9, 69.2%	14, 73.7%	26, 59.1%	.499^b^
Vis. disturb. (*n*, %)	53, 69.7%	6, 46.2%	15, 78.9%	32, 72.7%	.112^b^
Myalgia (*n*, %)	65, 85.5%	13, 100%	14, 73.7%	38, 86.4%	.112^b^
ED Diagn. (*n*, %)	11, 14.5%	2, 15.4%	1, 5.3%	8, 18.2%	.407^b^

a Data are shown as median (first quartile – third quartile).

b Pearson chi-squared test.

c Kruskal-Wallis test.

d Data are shown as median (first quartile – third quartile; minimum – maximum).

All the participants had > 12 years of education. SES = subjective educational and socio-economic status (scale range from 1 to 10 points). Annual income is reported on a 5-point scale (range from 10 to 50 thousand euros). Bla., Lat., Car., Afr. = Black, Latino, Caribbean or African; Months = months from diagnosis to assessment; BMI = body mass index; Confirm. Test = SARS-CoV-2 confirmed with antigen or PCR test; Hospit. = Hospitalisation; Vent. assist. = ventilatory assistance; RS = respiratory support; Metal. = metallic; Concent. = concentration; Disturb. = disturbance; Vis. = visual; Rec. = recurrent; ED diagn. = emotional disorder diagnosis based on clinical judgement (mood and/or anxiety disorders according to DSM-5 classification).

### 3.2. Differences based on the chronicity of the olfactory dysfunction

Table 3 presents the Kruskal-Wallis tests for the full sample of scores of the target variables and the statistic *H*, with its degrees of freedom and significance. Results showed that all the groups reported similar levels of memory complaints (MFE score and MFEA, MFER, and MFEC subscales, all *Ps* ≥ 0.20). Similarly, the groups did not differ in their self-reported anxious and depressive symptomatology (all *Ps* ≥ 0.09). The level of trait-anxiety was also similar in the groups (STAIT: *p* < 0.16).

**Table 3:** Mean ± standard deviation of the study variables and group comparisons in the full sample.

	AOD group	POD group	NOD group	Kruskal-Wallis test	
	(N = 22)	(N = 32)	(N = 74)	*H*-value (*df* = 2)	*p*-value
MFE	32.86 ± 7.96	33.88 ± 9.24	31.53 ± 11.65	0.99	0.61
MFEA	13.77 ± 3.19	15.03 ± 3.78	13.20 ± 4.85	3.23	0.20
MFER	3.09 ± 1.92	3.25 ± 2.14	3.14 ± 2.61	0.35	0.84
MFEC	16.00 ± 3.96	15.59 ± 4.36	15.19 ± 5.24	0.37	0.83
GADSA	6.41 ± 2.15	7.47 ± 1.48	6.80 ± 2.14	2.97	0.23
GADSD	5.23 ± 1.82	6.25 ± 1.41	6.01 ± 1.79	4.83	0.09
STAIT	4.64 ± 3.11	5.84 ± 2.53	5.70 ± 2.71	3.67	0.16

AOD group = acute olfactory dysfunction; POD group = persistent olfactory dysfunction; NOD group = absence of olfactory dysfunction; MFE = Memory Failures in Every-day life; MFEA = Memory of activities factor of the MFE; MFER = Recognition factor of the MFE; MFEC = Communication monitoring factor of the MFE; GADSA = Anxiety subscale of the Goldberg Anxiety and Depression Scale; GADSD = Depression subscale of the Goldberg Anxiety and Depression Scale; STAIT = Trait-Anxiety subscale of the State–Trait Anxiety Inventory.

**Figure 1:**
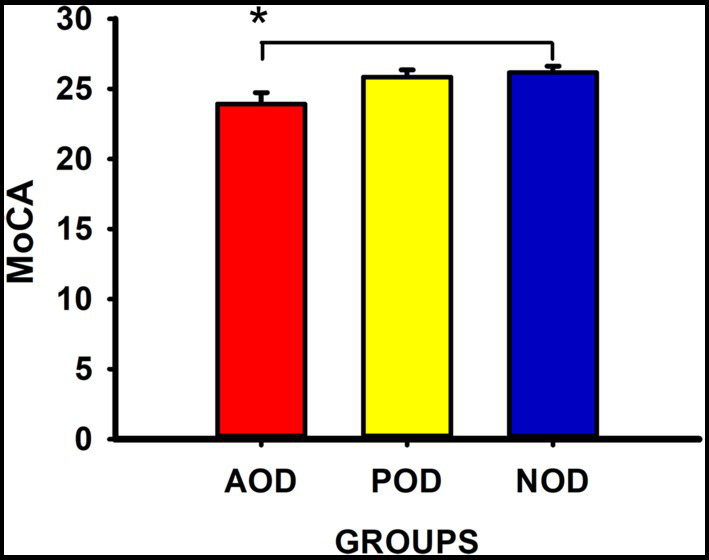
Montreal Cognitive Assessment (MoCA) scores (Mean ± SEM) in the groups. AOD group = acute olfactory dysfunction; POD group = persistent olfactory dysfunction; NOD group = absence of olfactory dysfunction. *statistically significant, *p* < 0.05.

Table 4 displays the descriptive statistics and the statistic *H*, with its degrees of freedom and significance, and the Kruskal-Wallis tests for the subsample in which we measured the general cognitive function (MoCA) and verbal episodic memory (PAL). In the same line as the results for the full sample, the groups did not differ in their self-rated levels of memory complaints, anxious and depressive symptomatology, or trait-anxiety (all *Ps* ≥ 0.39). The immediate and delayed ability to recall verbal episodic information was similar among the groups (PALIR and PALDR: *Ps* ≥ 0.27), and delayed recognition revealed no significant differences (PALDRe: *p* = 0.40). However, general cognitive ability was significantly different among the groups (MoCA: *p* = 0.02). The post-hoc multiple comparisons with Bonferroni correction showed that the general score of cognitive ability was lower in the participants with symptoms of acute olfactory dysfunction than in those with no olfactory symptoms at any time (*p* = 0.02, η^2^ = 0.08; Figure 1). The MoCA score did not differ between the participants of the POD group and the participants of the AOD or NOD groups (all *Ps* ≥ 0.34).

**Table 4:** Mean ± standard deviation of the study variables and group comparisons in the face-to-face assessed subsample.

	AOD group	POD group	NOD group	Kruskal-Wallis test	
	(N = 13)	(N = 19)	(N = 44)	*H*-value (*df* = 2)	*P*-value
MoCA	23.92 ± 2.93	25.84 ± 2.24	26.16 ± 3.03	7.46	**0.02**
PALIR	15.00 ± 4.88	14,47 ± 5.58	15.91 ± 7.28	0.83	0.66
PALDR	4.54 ± 1.90	5.26 ± 2.23	5.57 ± 2.29	2.64	0.27
PALDRe	23.31 ± 1.03	23.79 ± 0.42	23.11 ± 2.79	1.83	0.40
MFE	32.31 ± 8.17	32.74 ± 8.57	30.52 ± 12.26	0.43	0.81
MFEA	13,62 ± 3.57	14.74 ± 3.77	12.89 ± 5.19	1.79	0.41
MFER	3.08 ± 1.89	2.95 ± 2.15	2.89 ± 2.51	0.43	0.81
MFEC	15.62 ± 3.97	15.05 ± 3.94	14.75 ± 5.48	0.27	0.87
GADSA	6.00 ± 2.41	7.37 ± 1.50	6.80 ± 2.30	1.86	0.39
GADSD	5.38 ± 1.98	6.11 ± 1.45	6.07 ± 1.83	1.63	0.44
STAIT	4.62 ± 3.50	5.63 ± 2.14	5.52 ± 2.98	1.74	0.42

Significant difference is in bold. AOD group = acute olfactory dysfunction; POD group = persistent olfactory dysfunction; NOD group = absence of olfactory dysfunction; MoCA = Montreal Cognitive Assessment scale; PALIR = immediate recall score of the Paired-Associate Learning scale; PALDR = delayed recall score of the Paired-Associate Learning scale; PALDRe = recognition score of the Paired-Associate Learning scale; MFE = Memory Failures in Every-day life; MFEA = Memory of activities factor of the MFE; MFER = Recognition factor of the MFE; MFEC = Communication monitoring factor of the MFE; GADSA = Anxiety subscale of the Goldberg Anxiety and Depression Scale; GADSD = Depression subscale of the Goldberg Anxiety and Depression Scale; STAIT = Trait-Anxiety subscale of the State–Trait Anxiety Inventory.

### 3.3. Months from diagnosis to assessment, symptoms of anxiety and depression, trait-anxiety, and olfactory dysfunction as predictors of memory complaints

**Figure 2:**
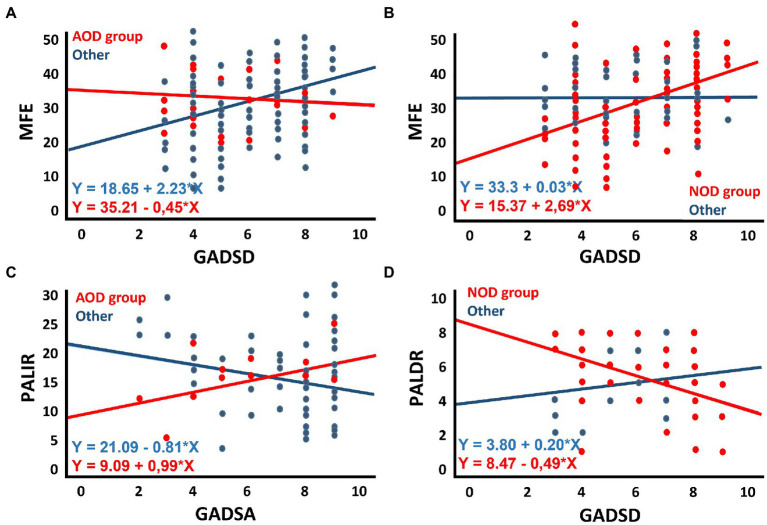
Scatter plots illustrating: **(A)** the interaction of depressive symptomatology (GADSD) in the groups of participants with or without acute olfactory dysfunction (with: AOD) in predicting memory complaints (MFE); **(B)** the interaction of depressive symptomatology (GADSD) in the groups of participants with or without symptoms of olfactory dysfunction (without: NOD) in predicting memory complaints (MFE); **(C)** the interaction of anxious symptomatology (GADSA) in the groups of participants with or without acute olfactory dysfunction (with: AOD) in predicting immediate ability to recall verbal episodic information (PALIR); **(D)** the interaction of depressive symptomatology (GADSD) in the groups of participants with or without olfactory dysfunction (without: NOD) in predicting delayed ability to recall verbal episodic information (PALDR).

Table 5 shows the results of the multiple regression analyses, including standardised betas, their significance, and the model’s general statistics. For simplification purposes, only the third block of each multiple regression is shown. The significant associations between scores of the MFE and main predictors are described below. The full scores of the MFE were predicted by anxious symptomatology and trait-anxiety in regression models that considered AOD (anxious symptomatology: *β* = 0.329, *p* = 0.001, 95% CI [0.733, 2.689]; trait-anxiety: *β* = 0.400, *p* < 0.001, 95% CI [0.803, 2.252]) and POD (anxious symptomatology: *β* = 0.264, *p* = 0.006, 95% CI [0.402, 2.341]; trait-anxiety: *β* = 0.316, *p* = 0.001, 95% CI [0.447, 1.939]) groups and after controlling for covariates. Thus, higher ratings on these variables were associated with higher subjective memory complaints. Also, considering depressive symptomatology as the main predictor, the full scores of the MFE were predicted by depressive symptomatology (*β* = 0.342, *p* = 0.001, 95% CI [0.919, 3.231]) by the AOD category (*β* = 0.609, *p* = 0.027, 95% CI [1.992, 31.765]) and the interaction term between depressive symptomatology and the AOD group (*β* = −0.555, *p* = 0.040, 95% CI [−5.391, −0.123]). Similarly, the MFE scores were predicted by the NOD category (*β* = −0.821, *p* = 0.007, 95% CI [−30.046, −4.736]) and the interaction term between depressive symptomatology and NOD group (*β* = 0.797, *p* = 0.016, 95% CI [0.479, 4.632]). Figures 2A,B depict these models graphically. Thus, depressive symptomatology was associated with memory complaints in participants who did not present olfactory dysfunction in the acute phase of the infection (Figure 2A), and this interaction was mainly explained by the contribution of the group’s NOD category, which comprised participants not suffering from olfactory dysfunction (Figure 2B).

**Table 5:** Multiple regressions predicting self-rated memory failures (MFE score), with months since COVID-19 onset, symptoms of anxiety and depression, trait-anxiety, and olfactory dysfunction as predictors.

Predictors	Criterion: MFE	β	*t*	*P*	95% CI	*R* ^2^	Adj *R*^2^	*F* change
Months,AOD	Age	−0.165	−1.763	0.080	−0.417, 0.024	0.081	0.035	1.024
	SES	−0.052	−0.564	0.574	−1.336, 0.743			
	Vent. Assist.	0.214	2.439	0.016	0.807, 7.768			
	Months	0.197	1.914	0.058	−0.010, 0.572			
	AOD	0.231	1.012	0.313	−6.118, 18.924			
	Months×AOD	−0.234	−1.012	0.313	−0.982, 0.318			
Months,POD	Age	−0.158	−1.658	0.100	−0.414, 0.037	0.087	0.041	0.898
	SES	−0.030	−0.341	0.733	−1.179, 0.832			
	Vent. Assist.	0.217	2.464	0.015	0.854, 7.842			
	Months	0.106	1.017	0.311	−0.143, 0.444			
	POD	−0.113	−0.498	0.620	−13.541, 8.102			
	Months×POD	0.217	0.948	0.345	−0.309, 0.876			
Months,NOD	Age	−0.147	−1.543	0.125	−0.400, 0.050	0.081	0.035	0.000
	SES	−0.055	−0.602	0.549	−1.367, 0.730			
	Vent. Assist.	0.222	2.501	0.014	0.924, 7.942			
	Months	0.143	0.997	0.321	−0.201, 0.610			
	NOD	−0.094	−0.412	0.681	−11.612, 7.609			
	Months×NOD	0.001	0.004	0.997	−0.514, 0.516			
GADSA, AOD	Age	−0.119	−1.409	0.161	−0.343, 0.058	0.140	0.098	1.073
	SES	−0.029	−0.331	0.741	−1.155, 0.824			
	Vent. Assist.	0.175	2.053	0.042	0.125, 6.883			
	GADSA	0.329	3.464	0.001	0.733, 2.689			
	AOD	0.337	1.210	0.229	−5.945, 24.624			
	GADSA×AOD	−0.286	−1.036	0.302	−3.392, 1.062			
GADSA, POD	Age	−0.114	−1.323	0.188	−0.341, 0.068	0.131	0.088	0.045
	SES	−0.019	−0.223	0.824	−1.081, 0.862			
	Vent. Assist.	0.183	2.110	0.037	0.226, 7.107			
	GADSA	0.264	2.802	0.006	0.402, 2.341			
	POD	−0.041	−0.100	0.920	−20.574, 18.594			
	GADSA×POD	0.089	0.212	0.832	−2.326, 2.885			
GADSA, NOD	Age	−0.110	−1.286	0.201	−0.334, 0.071	0.140	0.097	0.516
	SES	−0.034	−0.387	0.700	−1.186, 0.799			
	Vent. Assist.	0.181	2.093	0.038	0.196, 7.033			
	GADSA	0.190	1.324	0.188	−0.488, 2.460			
	NOD	−0.306	−0.971	0.334	−19.693, 6.733			
	GADSA×NOD	0.237	0.718	0.474	−1.168, 2.499			
GADSD, AOD	Age	−0.110	−1.300	0.196	−0.331, 0.069	0.145	0.103	4.295
	SES	−0.002	−0.026	0.979	−1.005, 0.978			
	Vent. Assist.	0.172	1.999	0.048	0.033, 6.839			
	GADSD	0.342	3.554	0.001	0.919, 3.231			
	AOD	0.609	2.245	0.027	1.992, 31.765			
	GADSD×AOD	−0.555	−2.073	0.040	−5.391, −0.123			
GADSD, POD	Age	−0.102	−1.178	0.241	−0.327, 0.083	0.119	0.076	0.805
	SES	0.001	0.013	0.990	−0.979, 0.991			
	Vent. Assist.	0.167	1.895	0.060	−0.149, 6.849			
	GADSD	0.271	2.798	0.006	0.481, 2.806			
	POD	0.388	1.045	0.298	−8.388, 27.143			
	GADSD×POD	−0.340	−0.897	0.371	−4.079, 1.535			
GADSD, NOD	Age	−0.100	−1.189	0.237	−0.319, 0.080	0.163	0.121	5.937
	SES	−0.004	−0.047	0.963	−1.011, 0.964			
	Vent. Assist.	0.164	1.922	0.057	−0.099, 6.683			
	GADSD	−0.011	−0.085	0.932	−1.691, 1.552			
	NOD	−0.821	−2.721	0.007	−30.046, −4.736			
	GADSD×NOD	0.797	2.437	0.016	0.479, 4.632			
STAIT, AOD	Age	−0.142	−1.698	0.092	−0.367, 0.028	0.173	0.132	2.819
	SES	−0.036	−0.419	0.676	−1.177, 0.766			
	Vent. Assist.	0.165	1.959	0.052	−0.035, 6.659			
	STAIT	0.400	4.175	<0.001	0.803, 2.252			
	AOD	0.313	1.937	0.055	−0.190, 17.548			
	STAIT×AOD	−0.270	−1.679	0.096	−2.857, 0.235			
STAIT, POD	Age	−0.117	−1.367	0.174	−0.342, 0.063	0.152	0.110	0.041
	SES	−0.025	−0.295	0.769	−1.099, 0.814			
	Vent. Assist.	0.163	1.882	0.062	−0.169, 6.694			
	STAIT	0.316	3.270	0.001	0.447, 1.939			
	POD	0.106	0.510	0.611	−7.379, 12.506			
	STAIT×POD	−0.044	−0.203	0.839	−1.739. 1.415			
STAIT, NOD	Age	−0.120	−1.431	0.155	−0.341, 0.055	0.180	0.139	2.702
	SES	−0.050	−0.592	0.555	−1.257, 0.678			
	Vent. Assist.	0.162	1.919	0.057	−0.103, 6.589			
	STAIT	0.166	1.333	0.185	−0.309, 1.579			
	NOD	−0.396	−2.135	0.035	−16.185, −0.609			
	STAIT×NOD	0.348	1.644	0.103	−0.214, 2.308			

The following covariates were included in the analyses: Age, SES and Ventilatory assistance. Significant *p*-values are underlined. *β* shows standardised values. Dichotomisation of the symptoms of olfactory dysfunction is 0 (no) and 1 (yes) for the following: AOD (acute olfactory dysfunction), POD (persistent olfactory dysfunction), NOD (absence of olfactory dysfunction); Covariates: Age, SES [subjective educational and socio-economic status (scale range from 1 to 10 points)], and Vent. Assist. [Ventilatory assistance: not applicable (0), enhanced respiratory support (1) and intubated (2)]. Months = months from diagnosis to assessment. GADSA = Anxiety subscale of the Goldberg Anxiety and Depression Scale; GADSD = Depression subscale of the Goldberg Anxiety and Depression Scale; STAIT = Trait-Anxiety subscale of the State–Trait Anxiety Inventory.

### 3.4. Months from diagnosis to assessment, symptoms of anxiety and depression, trait-anxiety, and olfactory dysfunction as predictors of general cognitive function

Table 6 shows the results of the multiple regression analyses and their main statistics. Only the third block of each multiple regression is shown. The significant associations between scores of the MoCA and main predictors are described below. Considering depressive symptomatology as the main predictor, and controlling for covariates, the scores of the MoCA were predicted by depressive symptomatology (*β* = −0.262, *p* = 0.042, 95% CI [−0.849, −0.015]) and by the AOD category (*β* = −0.69, *p* = 0.048, 95% CI [−10.568, −0.044]). The more symptomatology, the lower the scores in this test.

**Table 6:** Multiple regressions predicting general cognitive function (MoCA score), with months since COVID-19 onset, symptoms of anxiety and depression, trait-anxiety, and olfactory dysfunction as predictors.

Predictors	Criterion: MoCA	β	*t*	*P*	95% CI	*R* ^2^	Adj *R*^2^	*F* change
Months,AOD	Age	−0.075	−0.584	0.561	−0.103, 0.057	0.143	0.068	0.157
	SES	−0.124	−1.058	0.294	−0.602, 0.185			
	Vent. Assist.	−0.141	−1.199	0.235	−1.964, 0.490			
	Months	−0.136	−0.985	0.328	−0.166, 0.056			
	AOD	−0.366	−1.069	0.289	−8.059, 2.437			
	Months×AOD	0.140	0.397	0.693	−0.203, 0.303			
Months,POD	Age	−0.027	−0.206	0.838	−0.090, 0.073	0.094	0.014	0.040
	SES	−0.171	−1.442	0.154	−0.689, 0.111			
	Vent. Assist.	−0.135	−1.109	0.271	−1.981, 0.566			
	Months	−0.184	−1.261	0.212	−0.191, 0.043			
	POD	0.029	0.109	0.914	−3.371, 3.759			
	Months×POD	−0.053	−0.201	0.842	−0.232, 0.190			
Months,NOD	Age	−0.081	−0.610	0.544	−0.108, 0.057	0.131	0.054	0.203
	SES	−0.135	−1.148	0.255	−0.623, 0.168			
	Vent. Assist.	−0.160	−1.342	0.184	−2.085, 0.409			
	Months	−0.206	−1.186	0.240	−0.223, 0.057			
	NOD	0.090	0.317	0.752	−2.805, 3.863			
	Months×NOD	0.131	0.450	0.654	−0.142, 0.225			
GADSA, AOD	Age	−0.113	−0.964	0.338	−0.108, 0.037	0.137	0.062	0.224
	SES	−0.119	−1.012	0.315	−0.595, 0.194			
	Vent. Assist.	−0.133	−1.132	0.262	−1.922, 0.530			
	GADSA	−0.113	−0.875	0.384	−0.497, 0.194			
	AOD	−0.428	−1.298	0.199	−8.362, 1.770			
	GADSA×AOD	0.152	0.473	0.638	−0.579, 0.939			
GADSA, POD	Age	−0.11	−0.892	0.376	−0.111, 0.042	0.072	−0.008	0.816
	SES	−0.181	−1.533	0.130	−0.703, 0.092			
	Vent. Assist.	−0.143	−1.157	0.251	−2.036, 0.541			
	GADSA	0.004	0.029	0.977	−0.338, 0.348			
	POD	0.485	0.884	0.380	−4.076, 10.565			
	GADSA×POD	−0.506	−0.903	0.370	−1.437, 0.541			
GADSA, NOD	Age	−0.144	−1.177	0.243	−0.121, 0.031	0.106	0.028	0.04
	SES	−0.142	−1.204	0.233	−0.639, 0.158			
	Vent. Assist.	−0.16	−1.329	0.188	−2.102, 0.422			
	GADSA	0.001	0.004	0.997	−0.514, 0.516			
	NOD	0.298	0.759	0.450	−2.848, 6.347			
	GADSA×NOD	−0.085	−0.201	0.842	−0.714, 0.583			
GADSD, AOD	Age	−0.116	−1.022	0.310	−0.107, 0.034	0.180	0.109	1.457
	SES	−0.108	−0.947	0.347	−0.568, 0.202			
	Vent. Assist.	−0.098	−0.837	0.405	−1.746, 0.714			
	GADSD	−0.262	−2.067	0.042	−0.849, −0.015			
	AOD	−0.690	−2.012	0.048	−10.568, −0.044			
	GADSD×AOD	0.409	1.207	0.232	−0.355, 1.444			
GADSD, POD	Age	−0.103	−0.842	0.403	−0.108, 0.044	0.084	0.005	0.297
	SES	−0.174	−1.484	0.142	−0.691, 0.101			
	Vent. Assist.	−0.108	−0.857	0.395	−1.893, 0.756			
	GADSD	−0.117	−0.882	0.381	−0.632, 0.244			
	POD	0.267	0.548	0.586	−4.715, 8.284			
	GADSD×POD	−0.271	−0.545	0.588	−1.337, 0.764			
GADSD, NOD	Age	−0.137	−1.15	0.254	−0.117, 0.031	0.138	0.063	0.864
	SES	−0.129	−1.105	0.273	−0.609, 0.175			
	Vent. Assist.	−0.117	−0.966	0.337	−1.885, 0.655			
	GADSD	−0.025	−0.136	0.892	−0.642, 0.560			
	NOD	0.585	1.462	0.148	−1.251, 8.123			
	GADSD×NOD	−0.405	−0.929	0.356	−1.120, 0.408			
STAIT, AOD	Age	−0.121	−1.041	0.302	−0.110, 0.035	0.141	0.066	1.020
	SES	−0.134	−1.143	0.257	−0.619, 0.168			
	Vent. Assist.	−0.146	−1.23	0.223	−2.009, 0.476			
	STAIT	−0.05	−0.379	0.706	−0.317, 0.216			
	AOD	−0.435	−2.135	0.036	−6.474, −0.219			
	STAIT×AOD	0.207	1.01	0.316	−0.263, 0.802			
STAIT, POD	Age	−0.109	−0.905	0.368	−0.109, 0.041	0.107	0.029	3.433
	SES	−0.184	−1.59	0.117	−0.702, 0.079			
	Vent. Assist.	−0.166	−1.356	0.179	−2.155, 0.411			
	STAIT	0.136	1.075	0.286	−0.118, 0.394			
	POD	0.514	1.677	0.098	−0.653, 7.535			
	STAIT×POD	−0.579	−1.853	0.068	−1.313, 0.048			
STAIT, NOD	Age	−0.151	−1.248	0.216	−0.122, 0.028	0.107	0.029	0.025
	SES	−0.149	−1.256	0.213	−0.650, 0.148			
	Vent. Assist.	−0.176	−1.439	0.155	−2.198, 0.356			
	STAIT	0.023	0.122	0.903	−0.352, 0.398			
	NOD	0.19	0.769	0.444	−1.775, 4.004			
	STAIT×NOD	0.045	0.157	0.876	−0.437, 0.512			

The following covariates were included in the analyses: Age, SES and Ventilatory assistance. Significant *p*-values are underlined. *β* shows standardised values. Dichotomisation of the symptoms of olfactory dysfunction is 0 (no) and 1 (yes) for the following: AOD (acute olfactory dysfunction), POD (persistent olfactory dysfunction), NOD (absence of olfactory dysfunction); Covariates: Age, SES [subjective educational and socio-economic status (scale range from 1 to 10 points)], and Vent. Assist. [Ventilatory assistance: not applicable (0), enhanced respiratory support (1) and intubated (2)]. Months = months from diagnosis to assessment. GADSA = Anxiety subscale of the Goldberg Anxiety and Depression Scale; GADSD = Depression subscale of the Goldberg Anxiety and Depression Scale; STAIT = Trait-Anxiety subscale of the State–Trait Anxiety Inventory.

### 3.5. Months from diagnosis to assessment, symptoms of anxiety and depression, trait-anxiety, and olfactory dysfunction as predictors of the ability to recall verbal information

Tables 7, 8 present the results of the multiple regression analyses and their main statistics. Only the third block of each multiple regression is shown. Immediate recall and delayed recall are the criterion variables in Tables 7, 8, respectively. The significant associations are described below. Regarding anxious symptomatology (Table 7), the PALIR scores were predicted by the AOD category (*β* = −0.713, *p* = 0.037, 95% CI [−23.671, −0.730]) and the interaction term between anxious symptomatology and AOD group (*β* = 0.674, *p* = 0.044, 95% CI [0.051, 3.488]). Thus, anxious symptomatology was associated with the immediate ability to recall verbal information in participants who did not present olfactory dysfunction in the acute phase of the infection, the greater the number of anxious symptoms, the lower the PALIR score (Figure 2C). The months elapsed from diagnosis to assessment predicted the scores of the PALDR (Table 8) in regression models that considered AOD and NOD groups and after controlling for covariates (AOD: *β* = −0.325, *p* = 0.021, 95% CI [−0.173, −0.014]; NOD: *β* = −0.435, *p* = 0.013, 95% CI [−0.223, −0.027]). Thus, the more months elapsed the lower the scores in this test. Also, the scores of the PALDR were predicted by depressive symptomatology (*β* = −0.322, *p* = 0.015, 95% CI [−0.687, −0.078]) and by the AOD category (*β* = −0.818, *p* = 0.021, 95% CI [−8.370, −0.689]). The more symptomatology, the lower the scores in PALDR. Also, the PALDR scores were predicted by the NOD category, which included participants without olfactory dysfunction (*β* = 1.125, *p* = 0.005, 95% CI [1.460, 8.043]) and the interaction term between depressive symptomatology and NOD group (*β* = −1.048, *p* = 0.016, 95% CI [−1.199, −0.126]). Depressive symptomatology was associated with the delayed recall score in participants who did not present olfactory dysfunction, the more symptomatology, the lower the scores (Figure 2D).

**Table 7:** Multiple regressions predicting immediate ability to recall verbal episodic information (PALIR score), with months since COVID-19 onset, symptoms of anxiety and depression, trait-anxiety, and olfactory dysfunction as predictors.

Predictors	Criterion: PALIR	β	*t*	*P*	95% CI	*R* ^2^	Adj *R*^2^	*F* change
Months,AOD	Age	−0.122	−0.906	0.368	−0.262, 0.098	0.072	−0.010	0.607
	SES	0.01	0.084	0.934	−0.848, 0.922			
	Vent. Assist.	−0.02	−0.164	0.870	−2.990, 2.536			
	Months	−0.216	−1.504	0.137	−0.438, 0.062			
	AOD	−0.269	−0.755	0.453	−16.293, 7.346			
	Months×AOD	0.286	0.779	0.439	−0.347 0.792			
Months,POD	Age	−0.122	−0.946	0.347	−0.256, 0.091	0.129	0.053	3.158
	SES	−0.012	−0.104	0.918	−0.893, 0.804			
	Vent. Assist.	−0.052	−0.434	0.666	−3.288, 2.114			
	Months	−0.063	−0.44	0.661	−0.303, 0.194			
	POD	0.252	0.963	0.339	−3.912, 11.213			
	Months×POD	−0.464	−1.777	0.080	−0.847, 0.049			
Months,NOD	Age	−0.153	−1.132	0.262	−0.285, 0.079	0.096	0.016	0.644
	SES	0.035	0.291	0.772	−0.745, 1.000			
	Vent. Assist.	−0.04	−0.328	0.744	−3.202, 2.299			
	Months	−0.241	−1.361	0.178	−0.519, 0.098			
	NOD	−0.04	−0.138	0.890	−7.866, 6.845			
	Months×NOD	0.237	0.802	0.425	−0.242, 0.568			
GADSA, AOD	Age	−0.162	−1.362	0.178	−0.276, 0.052	0.106	0.028	4.22
	SES	0.027	0.230	0.819	−0.790, 0.996			
	Vent. Assist.	−0.001	−0.006	0.995	−2.784, 2.768			
	GADSA	−0.258	−1.972	0.053	−1.556, 0.009			
	AOD	−0.713	−2.122	0.037	−23.671, −0.730			
	GADSA×AOD	0.674	2.054	0.044	0.051, 3.488			
GADSA, POD	Age	−0.19	−1.525	0.132	−0.303, 0.041	0.058	−0.024	0.001
	SES	−0.008	−0.069	0.945	−0.922, 0.860			
	Vent. Assist.	−0.017	−0.139	0.890	−3.089, 2.686			
	GADSA	−0.105	−0.817	0.417	−1.082, 0.453			
	POD	−0.124	−0.224	0.823	−18.250, 14.564			
	GADSA×POD	0.014	0.025	0.980	−2.190, 2.244			
GADSA, NOD	Age	−0.176	−1.443	0.154	−0.291, 0.047	0.104	0.026	2.763
	SES	0.034	0.286	0.775	−0.760, 1.015			
	Vent. Assist.	−0.004	−0.036	0.971	−2.861, 2.759			
	GADSA	0.136	0.710	0.480	−0.738, 1.554			
	NOD	0.775	1.971	0.053	−0.121, 20.353			
	GADSA×NOD	−0.705	−1.662	0.101	−2.647, 0.241			
GADSD, AOD	Age	−0.177	−1.482	0.143	−0.288, 0.043	0.092	0.013	2.471
	SES	0.011	0.093	0.926	−0.860, 0.944			
	Vent. Assist.	0.018	0.149	0.882	−2.664, 3.093			
	GADSD	−0.259	−1.939	0.057	−1.925, 0.028			
	AOD	−0.606	−1.68	0.097	−22.684, 1.944			
	GADSD×AOD	0.56	1.572	0.121	−0.446, 3.765			
GADSD, POD	Age	−0.182	−1.48	0.143	−0.296, 0.044	0.076	−0.005	0.611
	SES	0.002	0.02	0.984	−0.876, 0.894			
	Vent. Assist.	0.022	0.176	0.861	−2.699, 3.220			
	GADSD	−0.191	−1.426	0.158	−1.679, 0.279			
	POD	−0.487	−0.994	0.323	−21.766, 7.284			
	GADSD×POD	0.391	0.782	0.437	−1.428, 3.268			
GADSD, NOD	Age	−0.188	−1.565	0.122	−0.296, 0.036	0.127	0.051	3.794
	SES	0.034	0.289	0.774	−0.751, 1.005			
	Vent. Assist.	0.026	0.216	0.830	−2.536, 3.151			
	GADSD	0.113	0.615	0.541	−0.931, 1.760			
	NOD	0.913	2.267	0.027	1.430, 22.411			
	GADSD×NOD	−0.855	−1.948	0.056	−3.380, 0.040			
STAIT, AOD	Age	−0.183	−1.491	0.141	−0.296, 0.043	0.048	−0.035	0.853
	SES	−0.013	−0.103	0.919	−0.969, 0.874			
	Vent. Assist.	−0.026	−0.210	0.834	−3.215, 2.603			
	STAIT	−0.014	−0.099	0.921	−0.655, 0.593			
	AOD	−0.200	−0.934	0.353	−10.752, 3.893			
	STAIT×AOD	0.199	0.924	0.359	−0.669, 1.824			
STAIT, POD	Age	−0.205	−1.648	0.104	−0.314, 0.030	0.052	−0.030	0.005
	SES	−0.016	−0.131	0.896	−0.955, 0.837			
	Vent. Assist.	−0.046	−0.363	0.718	−3.474, 2.404			
	STAIT	0.069	0.530	0.598	−0.431, 0.742			
	POD	−0.116	−0.366	0.715	−11.105, 7.659			
	STAIT×POD	−0.024	−0.073	0.942	−1.617, 1.503			
STAIT, NOD	Age	−0.211	−1.701	0.093	−0.318, 0.025	0.063	−0.018	0.512
	SES	0.01	0.084	0.933	−0.870, 0.946			
	Vent. Assist.	−0.039	−0.312	0.756	−3.363, 2.453			
	STAIT	0.155	0.817	0.417	−0.504, 1.203			
	NOD	0.315	1.245	0.217	−2.475, 10.687			
	STAIT×NOD	−0.212	−0.716	0.477	−1.469, 0.693			

The following covariates were included in the analyses: Age, SES and Ventilatory assistance. Significant *p*-values are underlined. *β* shows standardised values. Dichotomisation of the symptoms of olfactory dysfunction is 0 (no) and 1 (yes) for the following: AOD (acute olfactory dysfunction), POD (persistent olfactory dysfunction), NOD (absence of olfactory dysfunction); Covariates: Age, SES [subjective educational and socio-economic status (scale range from 1 to 10 points)], and Vent. Assist. [Ventilatory assistance: not applicable (0), enhanced respiratory support (1) and intubated (2)]. Months = months from diagnosis to assessment. GADSA = Anxiety subscale of the Goldberg Anxiety and Depression Scale; GADSD = Depression subscale of the Goldberg Anxiety and Depression Scale; STAIT = Trait-Anxiety subscale of the State–Trait Anxiety Inventory.

**Table 8:** Multiple regressions predicting delayed ability to recall verbal episodic information (PALDR score), with months since COVID-19 onset, symptoms of anxiety and depression, trait-anxiety, and olfactory dysfunction as predictors.

Predictors	Criterion: PALDR	β	*t*	*P*	95% CI	*R* ^2^	Adj *R*^2^	*F* change
Months,AOD	Age	−0.086	−0.665	0.508	−0.076, 0.038	0.140	0.064	0.377
	SES	0.072	0.615	0.540	−0.194, 0.368			
	Vent. Assist.	0.031	0.266	0.791	−0.760, 0.994			
	Months	−0.325	−2.356	0.021	−0.173, −0.014			
	AOD	−0.294	−0.857	0.395	−5.361, 2.140			
	Months×AOD	0.217	0.614	0.541	−0.125, 0.236			
Months,POD	Age	−0.054	−0.428	0.670	−0.068, 0.044	0.160	0.086	2.495
	SES	0.032	0.279	0.781	−0.236, 0.313			
	Vent. Assist.	0.018	0.156	0.876	−0.806, 0.943			
	Months	−0.222	−1.582	0.118	−0.144, 0.017			
	POD	0.318	1.237	0.220	−0.930, 3.966			
	Months×POD	−0.405	−1.580	0.119	−0.260, 0.030			
Months,NOD	Age	−0.085	−0.650	0.518	−0.077, 0.039	0.155	0.081	1.283
	SES	0.069	0.597	0.553	−0.195, 0.361			
	Vent. Assist.	0.019	0.161	0.873	−0.806, 0.947			
	Months	−0.435	−2.542	0.013	−0.223, −0.027			
	NOD	−0.165	−0.590	0.557	−3.037, 1.651			
	Months×NOD	0.324	1.133	0.261	−0.056, 0.202			
GADSA, AOD	Age	−0.189	−1.605	0.113	−0.095, 0.010	0.126	0.050	1.956
	SES	0.100	0.851	0.398	−0.164, 0.408			
	Vent. Assist.	0.031	0.260	0.796	−0.773, 1.004			
	GADSA	−0.256	−1.977	0.052	−0.498, 0.002			
	AOD	−0.657	−1.978	0.052	−7.310, 0.032			
	GADSA×AOD	0.454	1.398	0.166	−0.164, 0.935			
GADSA, POD	Age	−0.189	−1.518	0.134	−0.098, 0.013	0.057	−0.025	0.009
	SES	0.036	0.302	0.764	−0.245, 0.332			
	Vent. Assist.	0.025	0.205	0.838	−0.839, 1.032			
	GADSA	−0.128	−0.996	0.323	−0.373, 0.125			
	POD	0.032	0.057	0.954	−5.163, 5.469			
	GADSA×POD	−0.053	−0.093	0.926	−0.752, 0.685			
GADSA, NOD	Age	−0.204	−1.679	0.098	−0.100, 0.009	0.114	0.037	1.975
	SES	0.085	0.720	0.474	−0.183, 0.389			
	Vent. Assist.	0.018	0.153	0.879	−0.835, 0.974			
	GADSA	0.085	0.446	0.657	−0.286, 0.451			
	NOD	0.716	1.833	0.071	−0.268, 6.320			
	GADSA×NOD	−0.592	−1.405	0.164	−0.792, 0.137			
GADSD, AOD	Age	−0.201	−1.746	0.085	−0.097, 0.006	0.157	0.084	3.233
	SES	0.095	0.822	0.414	−0.165, 0.397			
	Vent. Assist.	0.059	0.492	0.624	−0.676, 1.119			
	GADSD	−0.322	−2.504	0.015	−0.687, −0.078			
	AOD	−0.818	−2.353	0.021	−8.370, −0.689			
	GADSD×AOD	0.617	1.798	0.077	−0.065, 1.249			
GADSD, POD	Age	−0.18	−1.466	0.147	−0.095, 0.015	0.080	0.001	0.738
	SES	0.048	0.411	0.682	−0.227, 0.345			
	Vent. Assist.	0.074	0.583	0.562	−0.676, 1.235			
	GADSD	−0.233	−1.742	0.086	−0.592, 0.040			
	POD	−0.432	−0.885	0.379	−6.771, 2.609			
	GADSD×POD	0.428	0.859	0.393	−0.432, 1.085			
GADSD, NOD	Age	−0.208	−1.788	0.078	−0.099, 0.005	0.179	0.108	6.07
	SES	0.09	0.794	0.430	−0.166, 0.385			
	Vent. Assist.	0.062	0.525	0.601	−0.657, 1.127			
	GADSD	0.146	0.818	0.416	−0.249, 0.595			
	NOD	1.125	2.879	0.005	1.460, 8.043			
	GADSD×NOD	−1.048	−2.464	0.016	−1.199, −0.126			
STAIT, AOD	Age	−0.202	−1.691	0.095	−0.099, 0.008	0.093	0.015	1.364
	SES	0.076	0.632	0.530	−0.199, 0.383			
	Vent. Assist.	0.021	0.174	0.862	−0.838, 0.999			
	STAIT	−0.128	−0.95	0.346	−0.291, 0.103			
	AOD	−0.400	−1.912	0.060	−4.530, 0.096			
	STAIT×AOD	0.246	1.168	0.247	−0.163, 0.624			
STAIT, POD	Age	−0.199	−1.590	0.116	−0.100, 0.011	0.045	−0.038	0.337
	SES	0.036	0.302	0.764	−0.247, 0.335			
	Vent. Assist.	0.009	0.070	0.944	−0.921, 0.989			
	STAIT	0.005	0.040	0.968	−0.187, 0.194			
	POD	0.128	0.404	0.687	−2.431, 3.666			
	STAIT×POD	−0.188	−0.581	0.563	−0.655, 0.359	0.082	0.002	0.606
STAIT, NOD	Age	−0.231	−1.877	0.065	−0.107, 0.003			
	SES	0.070	0.583	0.562	−0.206, 0.376			
	Vent. Assist.	0.002	0.014	0.989	−0.925, 0.938			
	STAIT	0.083	0.444	0.658	−0.213, 0.334			
	NOD	0.369	1.474	0.145	−0.551, 3.667			
	STAIT×NOD	−0.229	−0.779	0.439	−0.482, 0.211			

The following covariates were included in the analyses: Age, SES and Ventilatory assistance. Significant *p*-values are underlined. *β* shows standardised values. Dichotomisation of the symptoms of olfactory dysfunction is 0 (no) and 1 (yes) for the following: AOD (acute olfactory dysfunction), POD (persistent olfactory dysfunction), NOD (absence of olfactory dysfunction); Covariates: Age, SES [subjective educational and socio-economic status (scale range from 1 to 10 points)], and Vent. Assist. [Ventilatory assistance: not applicable (0), enhanced respiratory support (1) and intubated (2)]. Months = months from diagnosis to assessment. GADSA = Anxiety subscale of the Goldberg Anxiety and Depression Scale; GADSD = Depression subscale of the Goldberg Anxiety and Depression Scale; STAIT = Trait-Anxiety subscale of the State–Trait Anxiety Inventory.

## 4. Discussion

The present study is the first to determine the relevance of olfactory dysfunction, categorised as an acute or a persistent symptom of long-COVID, in the explanation of subjective and objective memory scores, general cognitive function, and mood disturbances. Results revealed no differences among the NOD, AOD, and POD groups in subjective memory complaints, depression and anxiety-related symptoms or levels of trait-anxiety. The three groups presented similar self-rated memory failures in every-day life regarding activities with either a prospective or retrospective memory component, recognition of places and people, and communication monitoring. They were also comparable in terms of their anxiety and depression symptomatology and trait-anxiety. Concerning the association of these scores in our long-COVID participants, higher depression and anxiety-related symptoms and level of trait-anxiety were associated with reporting more subjective memory failures. These associations were found after controlling for participants’ age, ventilatory assistance, and educational and socio-economic status. Our study revealed that the predictive value of the depressive symptoms for subjective memory failures is significantly stronger in individuals with no olfactory dysfunction. When assessing objective memory performance in a subsample of participants, those reporting olfactory dysfunction only during the acute phase of the disease presented lower scores in general cognition as assessed by MoCA than participants who had not experienced olfactory dysfunction. These lower scores were associated with depressive symptomatology after including covariates in the analyses. Self-reported memory failures were predicted by emotional symptoms in regression models that considered olfactory dysfunction. In addition, the association between depressive symptomatology and memory complaints was found specifically in the participants not suffering from olfactory dysfunction. Anxious symptomatology was negatively associated with the immediate ability to recall verbal information in participants who did not present olfactory dysfunction in the acute phase of the infection. The delayed recall of verbal information was predicted by depressive symptomatology in the regression model that considered the acute olfactory dysfunction. Besides, the more depressive symptomatology, the lower the delayed recall scores of the participants who did not present olfactory dysfunction. In general, these findings may contribute to further understanding of the neuropsychological and emotional aspects of long-COVID.

Compared to the NOD group of participants, the AOD group presented lower general cognition assessed with MoCA, which included an assessment of short-term memory and working memory, visuospatial abilities and orientation. Objective declarative memory, which is associated with hippocampal function (Squire and Dede, 2015), was not related to the persistence of olfactory dysfunction, and individuals with lower cognitive function had recovered from initial olfactory dysfunction. This is contrary to our hypothesis. We expected an association between the chronicity of olfactory dysfunction in long-COVID patients and cognitive and memory scores, due to a more deleterious effect of the virus on the olfactory system and limbic system regions (Doty, 2022). However, initial symptoms of COVID-19 are very relevant for long-term cognitive alterations. In this sense, recent research has shown that the symptoms during the initial phase of the disease, including olfactory dysfunction, could be determinants to produce brain alterations (Goehringer et al., 2022). Brain hypometabolism correlated with high inflammation and impaired cognition, assessed with MoCA, and was associated with a higher number of symptoms at the time of the initial infection (Goehringer et al., 2022). This hypometabolism affects frontal, insular and temporal cortices, all regions of the olfactory brain network (Guedj et al., 2021; Goehringer et al., 2022). However, this brain hypometabolism of frontal and insular cortices—regions strongly associated with initial olfactory dysfunction (Seubert et al., 2013)—is transient and does not persist over time (Martini et al., 2022). Nevertheless, the hippocampus and the amygdala also presented hypermetabolism that was long-lasting (Martini et al., 2022). This more persistent brain dysfunction could be responsible for the persistent cognitive deficits found in patients with recovered olfactory dysfunction. Brain plasticity could account for the different course of evolution of the olfactory symptoms. Brain connectivity of olfactory regions could explain inter-subject differences in the residual olfactory dysfunction found in patients post-infection (Esposito et al., 2022). We note that olfactory dysfunction was self-reported by the participants, and not objectively assessed. Therefore, this finding requires more research, as more studies are needed to elucidate the causes of recovered and persistent olfactory dysfunction and how they interact with cognitive function. The clinical course of olfactory loss after SARS-CoV-2 infection is not entirely understood, and the evidence of the duration and recovery of this symptom is inconsistent across studies (Agyeman et al., 2020; Santos et al., 2021). Studies are being made to elucidate how the initial severity of the dysfunction, viral load, concomitant symptoms, medical history, age, and sex are associated with persistent olfactory dysfunction (Saussez et al., 2021; Sehanobish et al., 2021; Chapurin et al., 2022; Tan et al., 2022). However, these variables are not yet thoroughly studied, and the results are contradictory.

Results revealed that the NOD, AOD, and POD groups were comparable in terms of their depression and anxiety-related symptoms and level of trait-anxiety. Anxiety and depression symptomatology and trait-anxiety were associated with reporting more subjective memory failures after controlling for participants’ age, ventilatory assistance, and educational and socio-economic status. However, only depression-related symptoms were associated with general cognitive function or memory when assessed objectively. Depression, followed by negative affect, such as higher levels of distress and anxiety, were the factors most highly related to memory complaints at all ages in normal population (Ponds et al., 1997; Clarnette et al., 2001; Reid and MacLullich, 2006; Zullo et al., 2021). Studies in long-COVID patients indicate a relationship between mood disorders and memory performance or complaints and persistent olfactory symptoms. However, these studies presented differences with our study. In the study of Voruz et al. (2022), subgroups of long-COVID patients, with a higher representation of males than in the present study, were made according to the severity of the acute illness, and a high prevalence of psychiatric symptoms and cognitive deficits were found regardless of the severity when compared to normative population. Long-term episodic memory assessed by Buschke test was impaired in the group with severe-acute symptoms and positively correlated with emotional apathy, but not with anxiety and depression. In this study, Voruz et al. (2022) objectively assessed persistent olfactory dysfunction using an olfaction test. In the group of patients with moderate olfactory symptoms, the olfactory dysfunction was associated with a diminished ability to recognise emotions, but not with memory function (Voruz et al., 2022). In addition, the study of Delgado-Alonso et al. (2022), which also used an objective measure of olfactory dysfunction, found an association between persistent olfactory dysfunction and delayed visual memory in a sample with a sex and age distribution comparable to the sample of our study. They also found that trait-anxiety moderately correlated with delayed verbal memory performance, and depression was not associated with objective cognitive scores. When assessing subjective memory complaints, neuropsychiatric scores were more relevant and, in agreement with our results, memory complaints were clearly associated with anxiety and depression in long-COVID participants (Almeria et al., 2020; Titze de Almeida et al., 2022). Interestingly, Almeria et al. (2020) also found an association of anosmia as an acute symptom non-objectively assessed with the working memory scores included in our assessment of cognition but not with delayed memory performance, as we found.

Olfactory dysfunction and older age are relevant predictors for the development of long-COVID (Brechbühl et al., 2021; Sudre et al., 2021). In our study we included participants’ age as covariate in regression models. Our participants’ age was below 65 years, so our sample is not aged. The association between age and better self-reported memory function during communication in studies using older samples of long-COVID patients than ours could be interpreted as impaired metacognition (Voruz et al., 2022). For this reason, it is important to consider age as a control variable in studies comparing subjective and objective memory performance in this population.

Self-report of memory by questionnaires offers an easily administered means of assessing the incidence of a range of memory failures and has been used in normal subjects (Papaliagkas et al., 2017) and patients suffering neurological diseases (Geffen et al., 1991). The MFE not only asked participants to recall instances of different forms of memory failure but also to rate the frequency with which they had occurred. This provides a more valid self-report than other methods, demanding more memory during their completion (Sunderland et al., 1984). However, based on regression analyses, self-reported memory failures are associated with depressive symptomatology, especially in long-COVID patients with no experience of olfactory dysfunction.

### 4.1. Limitations of the current study

This study presents several limitations. Firstly, we recruited voluntary participants. Therefore, moderately or slightly affected subjects were more prone to accept enrolment in the study. To some extent, this may influence our ability to generalise the findings to the total population with this syndrome, which includes subjects with severe long-COVID symptoms. Secondly, olfactory dysfunction, as well as other long-COVID symptoms, were evaluated 3–30 months after the acute phase of the COVID-19 infection by a subjective retrospective report. This method of assessment of olfactory dysfunction was also used in studies that included self-reported questionnaires to collect olfactory symptoms several months after the acute infection (Almeria et al., 2020; Helmsdal et al., 2022; Seeßle et al., 2022). However, the description of olfactory dysfunction was not provided by a standardised objective protocol and did not include an index of the severity of olfactory dysfunction. This report may be influenced not only by individuals’ subjective perception but also by memory function when reporting the presence of olfactory dysfunction at the acute phase of the infection. This limitation also applies to other reported symptoms at the time of assessment, which were not objectively assessed. Thirdly, we ignored participants’ pre-COVID memory cognitive and emotional state, so we cannot draw definite conclusions about a causal relationship between olfactory dysfunction and cognition. Finally, the questionnaire used to assess subjective memory function involves components of declarative episodic memory, working memory, language, attention, planning, and intentionality. The items of this questionnaire measure processes of recognition and recall of visual, verbal, and spatial information, prospective and retrospective memory, and executive control functions (Montejo et al., 2014). However, attention and executive function were not directly assessed by subjective questionnaires or objective tests in this study. We were mainly focused on declarative memory, as previous research has also found that this function is impaired in long-COVID patients (Damiano et al., 2022; Delgado-Alonso et al., 2022; Voruz et al., 2022; Llana et al., 2022b). However, attention and executive function are also significant processes affected in long-COVID patients (Delgado-Alonso et al., 2022).

## 5. Conclusion

The research shows that it is relevant to distinguish between participants on the basis of their olfactory dysfunction after SARS-CoV-2 infection. Olfactory dysfunction in the acute phase of the infection by COVID-19 is related to cognitive deficits in objective tests, and mood disturbances are associated with self-reported and objective memory. These findings may contribute to further understanding the neuropsychological and emotional aspects of long-COVID.

## Data availability statement

The raw data supporting the conclusions of this article will be made available by the authors, without undue reservation.

## Ethics statement

The studies involving human participants were reviewed and approved by Comité de ética en investigación Universitat Politècnica de València. The patients/participants provided their written informed consent to participate in this study.

## Author contributions

MM-L, MM and M-CJ conceived and planned the experiments. M-CJ acquired the funding and administrated the project. MM-L, MM, TL and SG-A carried out the experiments. MM-L, SG-A and TL contributed to the creation of the database. MM-L analysed the data. SG-A designed the graphic representation. MM, MM-L, VH, M-CJ and TL drafted the manuscript. All authors reviewed the manuscript, contributed to the article and approved the submitted version.

## Funding

This work was supported by the Conselleria d’Innovació, Universitats, Ciència i Societat Digital de la Generalitat Valenciana [GVA-COVID19/2021/025]; Gobierno de Aragón (Departamento de Ciencia, Universidad y Sociedad del Conocimiento) and FEDER “Construyendo Europa desde Aragón” for the research group with reference S31_20D.

## Conflict of interest

The authors declare that the research was conducted in the absence of any commercial or financial relationships that could be construed as a potential conflict of interest.

## Publisher’s note

All claims expressed in this article are solely those of the authors and do not necessarily represent those of their affiliated organizations, or those of the publisher, the editors and the reviewers. Any product that may be evaluated in this article, or claim that may be made by its manufacturer, is not guaranteed or endorsed by the publisher.
