# Supplementary material for: Association between olfactory dysfunction and mood disturbances with objective and subjective cognitive deficits in long-COVID
Source: Front Psychol. 2023 Feb 2;14:1076743. doi: 10.3389/fpsyg.2023.1076743 (PMC9932904; doi:10.3389/fpsyg.2023.1076743)
Supplement: Supplementary file 1 [file Table_1.docx]

Supplemental file

**S1.** Demographic information and clinical characteristics related to the COVID history of the full sample and the subsample and comparisons between them.

|  | Full sample | Subsample |  |  |
| --- | --- | --- | --- | --- |
|  | (N = 128) | (N = 76) | Test statistic | Significance: *P* |
| Sex (F, M; M%) | 114, 14; 10.9% | 68, 8; 10.5% | ꭓ^2^ (1) = 0.008 | >.999 |
| Age (Years)^a^ | 45.50 (40-51) | 46 (40-51) | *U* = 4779, *z* = -0.20 | .836 |
| SES^a^ | 7 (6-8) | 6 (5-8) | *U* = 4286, *z* = -1.44 | .149 |
| Annual income^a^ | 30 (20-40) | 30 (20-40) | *U* = 4640, *z* = -0.56 | .571 |
| Handedness (n, %) | |  | ꭓ^2^ (2) = 0.136 | >.999 |
| Right-hand | 118, 92.2% | 69, 90.8% |  |  |
| Left-hand | 6, 4.7% | 4, 5.3% |  |  |
| Ambidextrous | 4, 3.1% | 3, 3.9% |  |  |
| Ethnicity (n, %) |  |  | ꭓ^2^ (3) = 1.604 | .872 |
| White | 123, 96.1% | 73, 96.1% |  |  |
| Mixed ethnic groups | 1, 0.8% |  |  |  |
| Bla., Lat., Car., Afr. | 3, 2.3% | 3, 3.9% |  |  |
| Prefer not to say | 1, 0.8% |  |  |  |
| Months^b^ | 17 (11-21; 3-30) | 17 (11-20; 3-30) | *U* = 4610, *z* = -0.38 | .703 |
| BMI^a^ | 24.85 (21.82-29.39) | 25.53 (21.89-31.08) | *U* = 4659, *z* = -0.50 | .616 |
| Acute phase of COVID | |  |  |  |
| Confirm. Test | 110, 85.9% | 69, 90.8% | ꭓ^2^ (1) = 1.04 | .307 |
| Hospit. (n, %) | 34, 26.6% | 17, 22.4% | ꭓ^2^ (1) = 0.447 | .616 |
| Vent. assist. (n, %) |  |  | ꭓ^2^ (2) = 1.283 | .561 |
| Not applicable | 104, 81.3% | 64, 84.2% |  |  |
| Intubated | 6, 4.7% | 5, 6.6% |  |  |
| Enhanced RS | 18, 14.1% | 7, 9.2% |  |  |
| Long-COVID symptoms | |  |  |  |
| Sense of taste |  |  | ꭓ^2^ (1) = 0.101 | 0.977 |
| Ageusia (n, %) | 23, 18% | 15, 63.2% |  |  |
| Metal. taste (n, %) | 22, 17.2% | 13, 17.1% |  |  |
| Fatigue (n, %) | 122, 95.3% | 72, 94.7% | ꭓ^2^ (1) = 0.034 | >.999 |
| Brain fog (n, %) | 120, 93.8% | 70, 92.1% | ꭓ^2^ (1) = 0.202 | .776 |
| Lack concent. (n, %) | 127, 99.2% | 75, 98.7% | ꭓ^2^ (1) = 0.140 | >.999 |
| Sleep disturb. (n, %) | 104, 81.3% | 62, 81.6% | ꭓ^2^ (1) = 0.003 | >.999 |
| Nightmares (n, %) | 61, 47.7% | 37, 48.7% | ꭓ^2^ (1) = 0.20 | >.999 |
| Rec. fevers (n, %) | 35, 27.3% | 18, 23.7% | ꭓ^2^ (1) = 0.332 | .622 |
| Headache (n, %) | 89, 69.5% | 49, 64.5% | ꭓ^2^ (1) = 0.557 | .536 |
| Vis. disturb. (n, %) | 90, 70.3% | 53, 69.7% | ꭓ^2^ (1) = 0.008 | >.999 |
| Myalgia (n, %) | 108, 84.4% | 65, 85.5% | ꭓ^2^ (1) = 0.049 | .844 |
| ED Diagn. (n, %) | 22, 17.2% | 11, 14.5% | ꭓ^2^ (1) = 0.259 | .696 |

^a^Data are shown as median (first quartile – third quartile). ^b^Data are shown as median (first quartile – third quartile; minimum – maximum). ꭓ^2^ (dg) = Pearson chi-squared test (degrees of freedom). *U* = Mann-Whitney test. All the participants had >12 years of education.

SES = subjective educational and socio-economic status (scale range from 1 to 10 points). Annual income is reported on a 5-point scale (range from 10 to 50 thousand euros); Bla., Lat., Car., Afr. = Black, Latino, Caribbean or African; Months = months from diagnosis to assessment; BMI = body mass index; Confirm. Test = SARS-CoV-2 confirmed with antigen or PCR test; Hospit. = Hospitalization; Vent. assist. = ventilatory assistance; RS = respiratory support; Metal. = metallic; Concent. = concentration; Disturb. = disturbance; Vis. = visual; Rec. = recurrent; ED diagn. = emotional disorder diagnosis based on clinical judgement (mood and/or anxiety disorders according to DSM-5 classification).
